# Can Medical Chatbots Trigger Disinhibition and Encourage Health Information Disclosure?

**DOI:** 10.3390/healthcare14091218

**Published:** 2026-05-01

**Authors:** Abdallah Alsaad, Shadaid Alanezi, Loai Kayed B. Melhim, Adi Alsyouf

**Affiliations:** 1 Department of MIS, College of Business, University of Hafr Al Batin, Hafr Al Batin 39524, Saudi Arabia; 2 Department of Health Information Management and Technology, College of Applied Medical Sciences, University of Hafr Al Batin, Hafr Al Batin 39524, Saudi Arabia; alanezishd@uhb.edu.sa (S.A.); l.banimelhim@uhb.edu.sa (L.K.B.M.); 3 Department of Healthcare Management and Informatics, College of Health & Medical Sciences, Liwa University, Abu Dhabi 41009, United Arab Emirates; adi.alsyouf@lu.ac.ae; 4 Jadara University Research Center, Jadara University, Irbid 21110, Jordan

**Keywords:** medical chatbot, disinhibition, information disclosure, artificial intelligence

## Abstract

**Background/Objectives**: Medical chatbots are increasingly integrated into healthcare to facilitate patient communication, often under the assumption that they reduce stigma and foster the disclosure of sensitive information. However, empirical support for this effect remains inconsistent. Drawing on online disinhibition theory, this study introduces the concept of machine-mediated disinhibition (MMD) to examine whether chatbot consultations elicit greater disclosure than human-mediated or face-to-face interactions. **Methods**: A scenario-based, between-subjects experiment (n = 373) compared three modes: face-to-face, human-through-computer, and chatbot. **Results**: Results revealed no evidence of increased disinhibition in the chatbot condition. Conversely, participants were significantly less willing to disclose sensitive health information to chatbots than to humans. **Conclusions**: These findings suggest that in high-stakes healthcare contexts, trust-related concerns override disinhibition effects, leading to avoidance rather than openness. This study challenges the prevailing assumption that AI agents inherently facilitate disclosure and highlights the critical need for further research on trust in AI-mediated medical communication.

## 1. Introduction

The integration of chatbots has revolutionized numerous service areas, particularly in the medical field. Medical chatbots utilize artificial intelligence (AI) and natural language processing to provide a diverse range of healthcare services [1,2,3]. Chatbots provide expert medical information and guidance, enhancing accessibility and user engagement by accessing extensive medical databases and mimicking human reasoning and dialogue [4]. Proponents argue that these technologies have the potential to mitigate significant healthcare challenges, including disparities in access, resource limitations, and diagnostic complexities [4,5].

Nevertheless, the provision of suitable health advice and recommendations via chatbots is contingent upon the collection and analysis of comprehensive user health data, encompassing medical history, lifestyle habits, and physiological measurements [6,7,8]. The collected data enable tailored medical advice and enhance the efficacy of health recommendations. In general, disclosing sensitive health information often generates considerable hesitation among people due to a range of concerns, including privacy risks, potential misuse of personal data, and ethical issues related to confidentiality [9,10]. Moreover, the fear of stigma, especially in relation to mental health, sexual health, or chronic conditions, may further discourage individuals from sharing such information.

While these challenges are well covered in the context of traditional health informatics solutions [11,12,13], far less is known about how users behave when interacting with medical chatbots. Unlike static forms or human-to-human interactions, AI-driven systems present a novel set of psychological and technical factors that make human reactions different [14]. For instance, the perceived non-human nature of a chatbot may paradoxically reduce the fear of immediate social judgment and lead to more positive outcomes with respect to information disclosure [15,16,17,18,19]. Therefore, understanding why people may communicate differently in a chatbot context is a critical [14], underexplored area that requires focused investigation to build the next generation of trustworthy and widely adopted digital health tools.

Prior research in this area shows that participants who believed they were interacting with computerized communication mediums (e.g., virtual humans, virtual assistants, or chatbots) reported lower fear of self-disclosure, lower impression management, displayed their emotions more intensely, and were rated as being more willing to disclose information [15,16,17,20]. Prior research explains that user disclosure decisions are frequently guided by cognitive heuristics, or mental shortcuts, rather than a systematic cost-benefit analysis of each interaction [14,20,21,22]. These heuristics are typically activated by contextual and interface-related cues. A particularly relevant heuristic in this domain is the machine heuristic, which posits that machines are inherently more objective, precise, and secure than humans [21]. Consequently, interface cues that emphasize the automated nature of a chatbot are likely to foster greater user trust, particularly among individuals who strongly endorse this heuristic [21]. This cognitive shortcut (machine heuristic) leads users to perceive AI as a more reliable and secure recipient of sensitive information, thereby encouraging greater self-disclosure [20].

Prior research implicitly identifies key machine heuristics that facilitate information disclosure within the context of AI agents. These heuristics are rooted in the perception that machines are inherently non-judgmental and are a more secure repository for sensitive information [15,16,17,18,19,20]. While these studies offer valuable insights into how users interact with AI agents, they are often qualitative or have been conducted in contexts distinct from medical AI. More critically, they provide limited explanations of the underlying psychological mechanisms that facilitate a more open and honest disclosure of sensitive information in the context of using medical chatbots. Moreover, recent studies have challenged this optimistic view. Ref. [23] found that patients resist medical AI due to perceptions of reduced uniqueness and empathy. Ref. [24] documented “algorithm aversion,” where people distrust algorithms after seeing them err. Similarly, ref. [3] identified privacy concerns and lack of human interaction as key barriers to chatbot adoption in healthcare. Moreover, individuals may be less willing to disclose to AI due to concerns about trust, authenticity, data security, and algorithm aversion [23,24,25]. These contrasting findings suggest that the effect of chatbot mediation on disclosure is more nuanced than previously acknowledged, and that the healthcare context—with its high stakes and sensitivity—may activate different psychological mechanisms than those observed in low-stakes settings.

Consequently, the objective of the current study is to address these knowledge gaps by empirically examining whether interacting with a medical chatbot, in fact, increases information disclosure. Additionally, we examined whether a potential psychological mechanism, namely, Machine-Mediated Disinhibition (MMD), is associated with this phenomenon. Building upon the current literature on disinhibition [26,27,28,29], we define MMD as the tendency for individuals to experience a reduction in social restraint and no longer be controlled by concerns about self-presentation or judgment when interacting with a non-human system.

We argue that MMD is theoretically distinct from related constructs such as online disinhibition, reduced social presence, and perceived anonymity, and should be framed as a unique psychological state. While online disinhibition [30] describes the loosening of social barriers during digital communication between human actors, MMD is uniquely characterized by the non-human agency of the conversational partner. Unlike perceived anonymity [30], which concerns the user’s belief that their identity is shielded from a social “other,” MMD is rooted in the perception that the machine agent—as a non-conscious entity—is inherently incapable of moral judgment or social evaluation. Furthermore, MMD differs from reduced social presence; whereas social presence reflects the psychological “warmth” or human-likeness of a medium [31], MMD specifically captures the reduction in evaluative concern that arises from the absence of a human evaluator. Thus, MMD represents a specialized psychological state where the interaction partner’s ontological status as a machine, rather than the channel’s technical features alone, drives the disinhibitory effect. Despite the maturity of online disinhibition research in social media and forum contexts [29,32,33], its application to the unique, dyadic dynamics of human-AI interaction remains a significant scholarly blind spot. To address this, we situated our study within the mental health domain context. It is characterized by high sensitivity and stigma [34,35] and provides a critical testbed to examine whether machine-mediated openness can override the trust deficits inherent in high-stakes disclosure.

## 2. Materials and Methods

### 2.1. Procedures

This study employed a scenario-based, between-subjects experiment to examine whether participants’ willingness to disclose sensitive health information and their level of machine-mediated disinhibition varied across three distinct conditions: Medical Chatbot, Human-through-Computer, and Face-to-Face provider.

The three experimental conditions were systematically selected to represent the current spectrum of healthcare interactions. The face-to-face condition served as the primary baseline, representing the traditional gold standard of medical consultation characterized by high social presence and nonverbal synchrony. The human-mediated computer interaction condition was designed to isolate the effect of the communication medium, modeling modern telehealth practices by removing physical presence while maintaining human agency. Finally, the medical chatbot condition allowed for a targeted examination of Machine-Mediated Disinhibition (MMD) by introducing a non-human interlocutor.

To maintain high internal validity and prevent conceptual confounding between the human-mediated computer interaction and medical chatbot condition conditions, we strictly operationalized agent identity: (1) the human condition featured a doctor (“Dr. Johnny Doe”) with a human profile icon, and (2) the chatbot condition featured an AI agent (“MindGuideBot”) explicitly labeled as an AI system with a machine icon. Crucially, both conditions utilized identical scripts, ensuring that any observed differences in disclosure were attributable solely to the perceived identity of the partner rather than the content of the interaction. Together, these conditions provide a robust framework for disentangling the effects of medium and agency in sensitive healthcare contexts.

**Figure A1:**
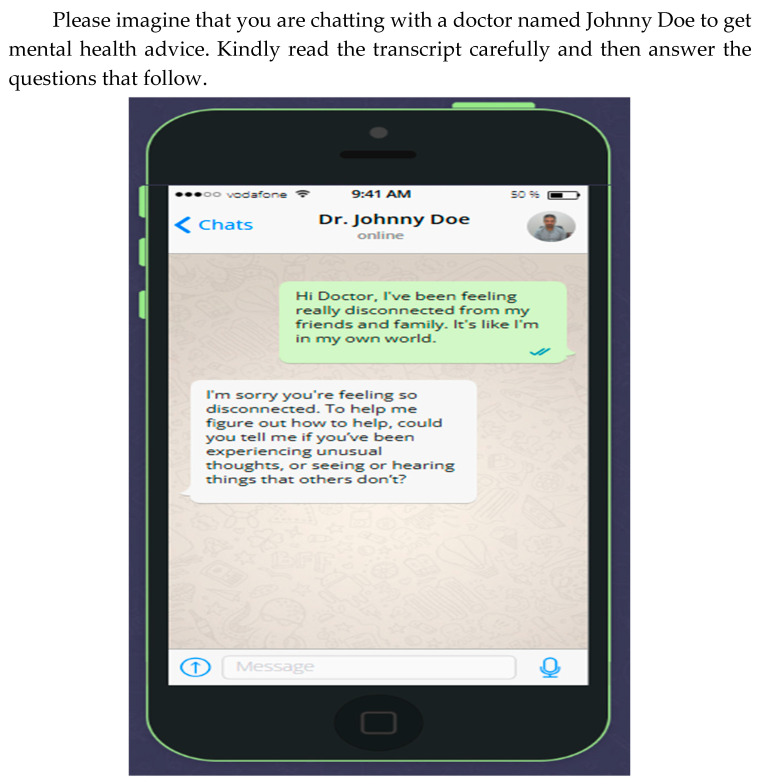
Human through Computer Condition.

**Figure A2:**
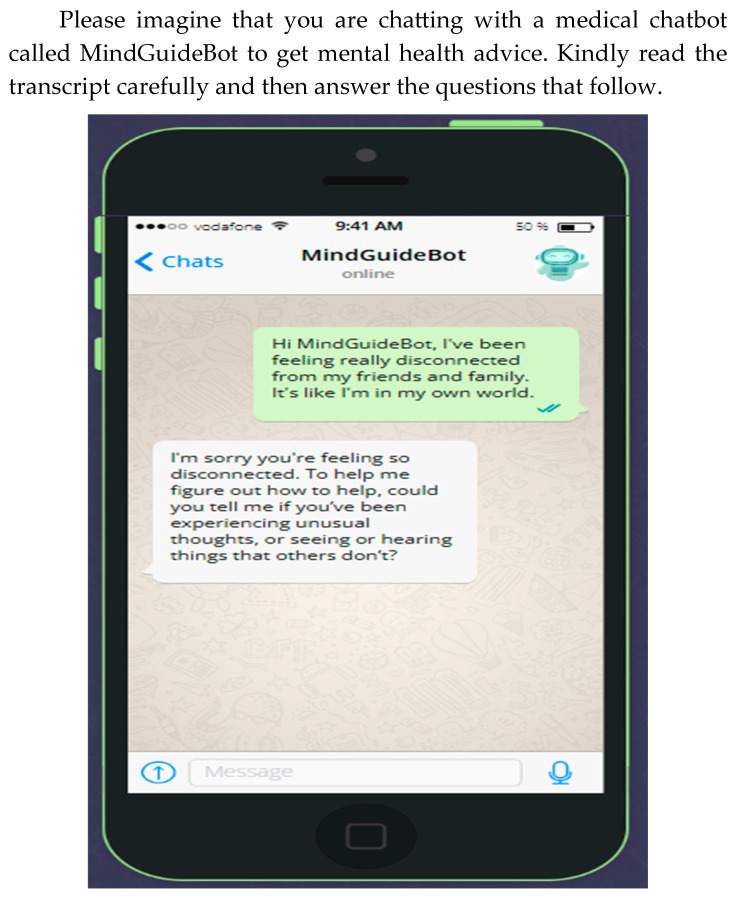
Medical Chabot Condition.

**Figure A3:**
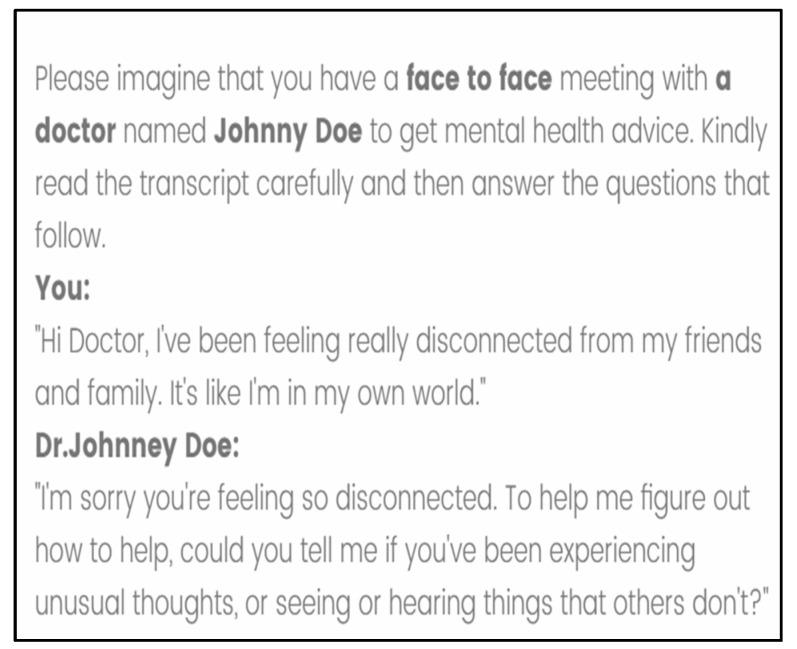
Face to face condition.

Participants were asked to imagine they were having a mental health consultation, prompted by an opening line: “I’ve been feeling really disconnected from my friends and family. It’s like I’m in my own world.” The task was supported by a chat transcript that was identical across all three groups; only the source of the interaction varied. The final message in the conversation prompted participants to disclose private and sensitive health information by asking, “Do you ever see or hear things that others don’t?” In the Chatbot and Human-through-Computer conditions, participants viewed a screenshot of a smartphone displaying the chat exchange. In the human-through-computer condition, the interface included a human-like icon representing the doctor, while in the chatbot condition, it depicted a medical chatbot, identified as “MindGuideBot.” In the Face-to-Face Condition, participants were not provided with a visual aid and were instead instructed to imagine the conversation taking place in person. To ensure the scenario’s ecological validity, it was subjected to a formal validation process by three domain experts. These experts assessed the scenario for face validity, realism, and clarity, ensuring the health context was accurately represented and the instructions were unambiguous. For full details, see Figure A1, Figure A2 and Figure A3 in Appendix A.

### 2.2. Ethical Considerations

Ethical approval for this study was granted by the Permanent Committee for Scientific Research Ethics at the University of Hafr Albatin, Saudi Arabia (approval number: HPO-05-FT-25/15). All procedures involving human participants were conducted in accordance with the committee’s guidelines to ensure the protection of the participants’ rights, privacy, and well-being. Before data collection, informed consent was obtained from all participants through a consent form that clearly outlined the purpose of the study, the voluntary nature of participation, and the measures taken to ensure confidentiality and anonymity. Respondents received an equivalent of €14 per hour for completing the survey.

### 2.3. Participants

A total of 373 participants were recruited through Prolific, an online participant recruitment platform widely recognized for providing high-quality and demographically diverse samples for behavioral research [36,37]. Of the sample, 190 participants were male (50.9%) and 183 were female (49.1%), with a mean age of 43.62 years (SD = 12.82; range = 20–77). Regarding educational attainment, the largest subgroup reported holding a bachelor’s degree (n = 142, 38.1%), followed by graduate or professional degrees (n = 71, 19.0%), some college or associate degree (n = 67, 18.0%), high school diploma (n = 61, 16.4%), and doctoral or professional doctorate (n = 4, 1.1%). An additional 27 participants (7.2%) reported completing some graduate work without earning a degree. Participants were randomly assigned to experimental conditions, with 141 in the human-through-computer condition, 98 in the face-to-face human condition, and 134 in the chatbot condition.

### 2.4. Measures

Two key variables were measured in this study: MMD and users’ willingness to disclose private health information. MMD was assessed using an adapted version of the Online Disinhibition Scale developed by [38]. An example item is: “I feel less constrained to discuss my mental health issues with this [chatbot/doctor]” The adapted scale demonstrated high internal consistency (Cronbach’s α = 0.91). Users’ willingness to disclose private health information was measured with three items adapted from [36]. A sample Item includes: “How likely are you to disclose your private mental health information with this [chatbot/doctor]?” The adapted scale demonstrated high internal consistency (Cronbach’s α = 0.98). A manipulation check was conducted at the end of the scenarios. Participants rated the statement, “I think I had a chat with a machine-based agent for medical advice” on a 7-point Likert scale to assess their perception of the agent (1 = strongly disagree, 7 = strongly agree).

To establish construct validity, a confirmatory factor analysis (CFA) was conducted using lavaan (Version 0.6-18) with maximum likelihood estimation to validate the two-factor measurement model (MMD: six items; Users’ willingness to disclose private health information: three items). The model demonstrated acceptable fit: χ^2^(26) = 141.75, *p* < 0.001; Comparative Fit Index (CFI) = 0.972; Tucker-Lewis Index (TLI) = 0.961; Standardized Root Mean Square Residual (SRMR) = 0.040 [37].

All factor loadings were significant (*p* < 0.001). MMD loadings ranged from 0.674 to 0.924, and Users’ willingness to disclose private health information loadings ranged from 0.977 to 0.981. The AVE for MMD was 0.64 and for Users’ willingness to disclose private health information was 0.96.

The correlation between MMD and Users’ willingness to disclose private health information was r = 0.883 (95% CI [0.856, 0.909]). To formally test discriminant validity, we compared the unconstrained two-factor model against a constrained model where the correlation was fixed to 1.0. The chi-square difference test was significant (Δχ^2^(1) = 356.99, *p* < 0.001), indicating that the two-factor model fits significantly better than a single-factor model. This confirms that MMD and Users’ willingness to disclose private health information, while correlated, are empirically distinct constructs. Conceptually, MMD represents the psychological state of reduced social restraint (the mechanism), whereas Disclosure represents the behavioural willingness to share information (the outcome). A strong correlation between a mechanism and its outcome is theoretically expected.

## 3. Results

**Figure 1:**
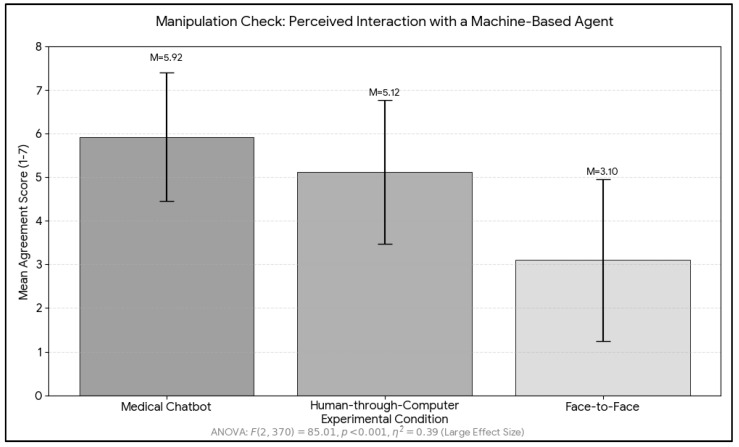
Mean of manipulation check across conditions.

To assess whether participants correctly perceived the nature of the agent they were interacting with, we conducted a manipulation check using the item “I think I had a chat with a machine-based agent for medical advice.” The means were in the expected direction for the three conditions. As shown in Figure 1, participants in the chatbot condition reported the highest agreement (M = 5.92, SD = 1.47), followed by those in the human through computer condition (M = 5.12, SD = 1.65), whereas participants in the face-to-face human condition reported the lowest agreement (M = 3.10, SD = 1.86). A one-way ANOVA revealed a significant effect of condition on participants’ perception of interacting with a machine-based agent (F (2, 370) = 85.01, *p* < 0.001, η^2^ = 0.39), indicating a large effect size. Post-hoc comparisons showed that all pairwise differences were significant (*p* < 0.001).

**Figure 2:**
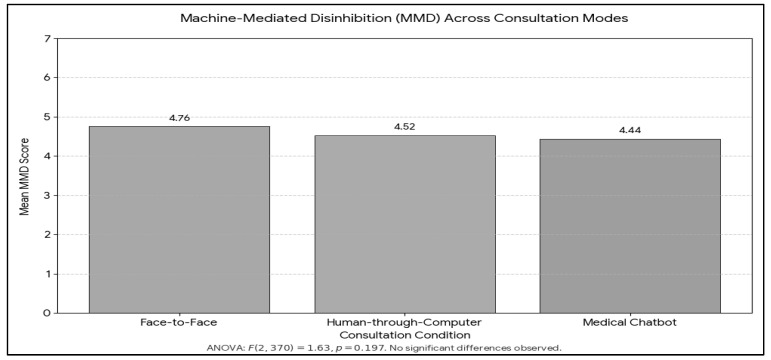
Mean of MMD across conditions.

ANOVA results revealed no significant differences in MMD across conditions (F(2, 370) = 1.63, *p* = 0.197). The mean scores were relatively close: human through computer condition (M = 4.52), chatbot condition (M = 4.44), and face-to-face human condition (M = 4.76). Post-hoc Scheffé comparisons confirmed that none of the pairwise differences were statistically significant. Figure 2 depicts the results. However, an ANCOVA was then conducted with age and gender as covariates to control for potential demographic influences. The effect of condition remained non-significant, F(2, 368) = 1.66, *p* = 0.192, partial η^2^ = 0.009. Neither age, F(1, 368) = 1.15, *p* = 0.285, partial η^2^ = 0.003, nor gender, F(1, 368) = 0.28, *p* = 0.598, partial η^2^ = 0.001, significantly predicted MMD.

These findings suggest that the mode of interaction did not meaningfully affect participants’ sense of being less constrained, judged, or pressured in their communication. Contrary to expectations, interacting with a chatbot did not produce greater disinhibition compared to the human-through-computer or face-to-face human conditions. This indicates that the MMD may be more stable across contexts than theorized or that other factors (e.g., task sensitivity and prior experience with AI) play a stronger role in shaping disinhibition.

**Figure 3:**
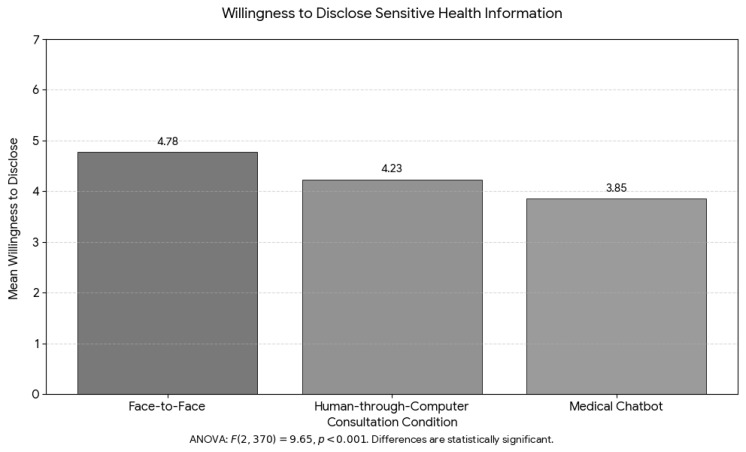
Users’ willingness to disclose sensitive health information across conditions.

In contrast, a one-way ANOVA revealed a significant effect of condition on disclosure, F(2, 370) = 9.65, *p* < 0.001, η^2^ = 0.050, representing a medium effect size. As shown in Figure 3, participants in the face-to-face condition reported the highest willingness to disclose (M = 4.78, 95% CI [4.52, 5.04]), followed by human-through-computer (M = 4.23, 95% CI [3.98, 4.48]), and chatbot (M = 3.85, 95% CI [3.58, 4.12]). Post-hoc pairwise comparisons with Bonferroni correction showed that disclosure was significantly higher in the face-to-face condition compared to chatbot (mean difference = 0.93, 95% CI [0.42, 1.43], *p* < 0.001, Cohen’s d = 0.59) and compared to human-through-computer (mean difference = 0.55, 95% CI [0.05, 1.06], *p* = 0.031, Cohen’s d = 0.29). The difference between chatbot and human-through-computer was not significant (mean difference = 0.37, 95% CI [−0.09, 0.83], *p* = 0.150, Cohen’s d = 0.22).

To examine whether this effect remained robust after controlling for demographic variables, an ANCOVA was conducted with age and gender as covariates. The effect of condition remained significant, F(2, 368) = 9.62, *p* < 0.001, partial η^2^ = 0.050. Neither age, F(1, 368) = 0.68, *p* = 0.410, partial η^2^ = 0.002 nor gender, F(1, 368) = 0.04, *p* = 0.849, partial η^2^ = 0.000, significantly predicted disclosure.

## 4. Discussion and Implications

Prior research has paradoxically suggested that the nonhuman nature of chatbots can reduce the fear of immediate social judgment, leading to more positive outcomes for information disclosure [15,16,17,18,19]. Although not always explicitly labeled as such, an online disinhibition effect has been observed in human-computer and human-AI interactions [15,16,17,20,38,39,40,41,42,43,44]. These studies consistently show that participants who believe they are interacting with a computerized communication medium, such as a virtual human, virtual assistant, or chatbot, report lower fear of self-disclosure and reduced impression management. They also tend to display their emotions more intensely and are rated as being more willing to disclose private information [15,16,17,20]. However, a contrasting literature suggests that in high-stakes contexts like healthcare, trust deficits and algorithm aversion may override disinhibition effects [23,24]. Many of these findings come from qualitative studies or research conducted outside the specific context of medical AI, which is a key area for future exploration.

This study addresses this gap by using a scenario-based, between-subjects experiment to compare whether participants were more willing to disclose sensitive health information and showed greater MMD when interacting with a medical chatbot versus a human provider (both face-to-face and through a computer interface). Contrary to our expectations, the participants did not report higher MMD when interacting with the chatbot. Furthermore, participants showed the highest willingness to disclose information in the face-to-face condition, followed by the human-through-computer condition, with the chatbot condition showing the lowest willingness to disclose information.

While our findings challenge the elementary conclusions of some qualitative studies, they align with a broader stream of research emphasizing ambivalence toward medical AI use [23,24,25]. However, beyond general aversion to AI, our results suggest that resistance to disclosing sensitive health information may be better understood through three complementary lenses: trust, privacy concerns, and perceived authenticity [12,45,46,47,48,49].

First, from a trust-based perspective, individuals may hesitate to disclose sensitive information not merely because the agent is artificial, but because AI systems are often perceived as lacking accountability, moral agency, and relational responsibility [50,51]. In high-stakes domains such as healthcare, trust is not only cognitive (i.e., competence-based) but also relational and affective [52]. Even if medical chatbots are perceived as technically competent, the absence of human intentionality and ethical reciprocity may limit users’ willingness to become vulnerable, thereby suppressing disclosure.

Second, a privacy-based explanation suggests that disclosure resistance is driven by heightened concerns over data security, surveillance, and secondary data use [9,10,53]. Unlike human physicians, AI systems are often associated with opaque data practices and large-scale data aggregation. This perceived loss of control over personal information may activate risk-avoidance mechanisms [10], particularly when the information is highly sensitive. In this sense, the healthcare context amplifies privacy calculus processes [53], where perceived risks outweigh potential benefits, thus counteracting any disinhibition effects.

Third, from an authenticity perspective, users may perceive AI-mediated interactions as lacking genuine understanding, empathy, and experiential grounding [52]. Authenticity plays a critical role in health communication, where patients often seek not only accurate information but also validation and emotional resonance [54]. The perception that AI responses are simulated rather than genuinely “felt” [55,56,57], may undermine the depth of interaction and reduce users’ willingness to share personal or stigmatized information.

In these lights, our results underscore the paradox that although chatbots can theoretically lower impression-management concerns, they may simultaneously evoke new barriers tied to technological uncertainty and a perceived lack of trust and empathy.

Our findings also point to an important boundary condition for the online disinhibition effect: the context of communication. In high-stakes, sensitive settings like healthcare, disinhibition alone may not be enough to encourage disclosure if the communication medium fails to provide adequate motivation. From a social presence perspective [58,59], chatbots lack the nonverbal immediacy and relational warmth of human providers, which may explain why participants were most willing to disclose information in face-to-face interactions. Similarly, media richness theory suggests that the leaner communication cues offered by chatbots, and computer-mediated human interactions are less effective for conveying authenticity [58], thus weakening disclosure intentions.

## 5. Practical Implications

Our results demonstrate that patients are less willing to disclose sensitive health information to chatbots compared to human providers (face-to-face or computer-mediated). To address this gap, we offer the following recommendations. First, prioritize trust-building before disclosure. Since trust deficits likely explain lower disclosure (as our mediation analysis suggested), chatbots should begin consultations by establishing credibility—explaining their purpose, data security measures, and limitations. A brief trust-building preamble may reduce initial resistance. Second, mimic human interaction patterns. Our finding that face-to-face consultations produced the highest disclosure suggests that human-like qualities matter. Chatbots should use conversational, non-scripted language, avoid repetitive phrasing, and incorporate natural pauses and affirmations (e.g., “I understand,” “Tell me more”). Third, offer a human escalation option. Given that participants preferred human interaction for disclosure, chatbots should not replace humans entirely. Instead, design hybrid systems where chatbots handle initial intake but seamlessly transfer to human providers when patients show hesitation or when sensitive topics arise. Fourth, be transparent about AI limitations. Rather than pretending to be human, chatbots should honestly state what they can and cannot do. Transparency about AI capabilities may paradoxically increase trust, as patients appreciate honesty about limitations. Future research should test these specific design features experimentally to determine which most effectively increases disclosure.

## 6. Limitation and Future Research

This study had several limitations. First, it did not control for important variables such as perceived risk and privacy concerns, which are crucial in a sensitive context such as healthcare. Future research should integrate these variables in their model for more robust results. Second, the use of a standardized transcript across all conditions, while methodologically clean for isolating agent identity, necessarily sacrifices some ecological validity. Real chatbot interactions involve dynamic, responsive exchanges with different interaction styles (e.g., structured questions, limited empathy, predictable phrasing) that may independently affect disclosure. We acknowledge this design trade-off between internal and external validity. Future research should include conditions with actual chatbot interactions or vary response tone (empathetic vs. neutral). Accordingly, a future experiment conducted with real patients could offer deeper insights into this issue and provide results with greater external validity. Future research should consider these factors for a better understanding of their influence on user behavior.

While our findings show that the general online disinhibition effect does not fully explain why people behave differently with medical AI, it is clear that other factors are at play. Culture may play a significant role, as societal norms regarding privacy and doctor-patient relationships vary widely. Future research should also explore the specific conditions under which a medical chatbot might actually lead to disinhibition and increased information disclosure. This could involve examining different chatbot designs, levels of anthropomorphism, or specific types of health-related tasks in which users might feel more comfortable sharing information with an AI.

## 7. Conclusions

This study provides evidence that the effects of chatbot-mediated communication in healthcare cannot be understood solely through the lens of online disinhibition. While prior research suggests that the nonhuman nature of chatbots may reduce fears of social judgment and encourage openness, our results showed that participants were not more disinhibited when interacting with a chatbot, and they were significantly less willing to disclose sensitive health information compared to face-to-face communication, with disclosure to chatbots being the lowest of all.

## Data Availability

Data cannot be shared publicly due to restrictions imposed by the study’s founder and the responsible institution. Access to the data requires prior approval from the founder. Interested researchers may request access by contacting the corresponding author.

## Appendix A

**Measures of the study**

A. Willingness to disclose private health information

1. How likely are you to disclose your private mental health information to this [doctor/chatbot].
2. How probable is it that you would disclose your private mental health information to this [doctor/chatbot]?
3. How willing are you to disclose your private mental health information to this [doctor/chatbot].

B. Machine-mediate disinhibition

1. I feel at ease discussing my mental health issues with this [doctor/chatbot].
2. I feel less constrained to discuss my mental health issues with this [doctor/chatbot].
3. I feel less concerned about being judged by this [doctor/chatbot].
4. I feel free to express myself bluntly with this [doctor/chatbot].
5. I feel less social pressure with this [doctor/chatbot].
6. I feel easy to express my true feelings or thoughts with this [doctor/chatbot].
